# Association of Blood Biomarkers Cell-Free DNA (cfDNA) and High-Sensitivity C-Reactive Protein (hsCRP) With Stroke Severity and Outcome: A Prospective Observational Study

**DOI:** 10.7759/cureus.82775

**Published:** 2025-04-22

**Authors:** Atulabh Vajpeyee, Monali Hiwarkar, Manisha Vajpeyee, Onjal K Taywade

**Affiliations:** 1 Neurosciences, Pacific Medical University, Udaipur, IND; 2 Anatomy, All India Institute of Medical Sciences, Jammu, Jammu, IND; 3 Reproductive Medicine and Research, Pacific Medical University, Udaipur, IND; 4 Biochemistry, All India Institute of Medical Sciences, Jammu, Jammu, IND

**Keywords:** acute ischemic stroke, cell-free dna, high-sensitivity c-reactive protein, mrs, nihss

## Abstract

Background

Stroke remains a leading cause of disability and mortality worldwide, with a rising incidence in India. Early identification of patients at risk for severe stroke and poor outcomes is crucial for timely intervention. Despite advancements, current diagnostic tools lack sufficient sensitivity and specificity for early prognostic stratification. Emerging evidence highlights cell-free DNA (cfDNA), a marker of cellular injury, and high-sensitivity C-reactive protein (hsCRP), an inflammatory marker, as promising candidates. These biomarkers were selected over others due to their robust association with tissue damage and inflammation, two pivotal mechanisms in stroke pathophysiology. This study aimed to assess the prognostic value of cfDNA and hsCRP in acute ischemic stroke patients and their association with stroke severity and outcomes.

Methods

This prospective observational study included 54 acute ischemic stroke patients admitted within 12 hours of symptom onset. Clinical assessments were performed using the National Institutes of Health Stroke Scale (NIHSS) at admission and the modified Rankin Scale (mRS) at three months to evaluate stroke severity and outcomes. Blood samples were collected to measure cfDNA and hsCRP levels. Correlation analyses were conducted to evaluate the association between biomarkers and stroke severity (NIHSS) and outcomes (mRS). Receiver operating characteristic (ROC) curve analysis determined optimal biomarker thresholds and logistic regression analysis identified independent predictors of poor neurological outcomes (mRS ≥ 3).

Results

The median age of the cohort was 61 years, with a mean of 61.6 ± 16.1 years, and 68.5% were male. cfDNA showed significant correlations with NIHSS (ρ = 0.222, p = 0.040) and mRS (ρ = 0.396, p = 0.002), while hsCRP correlated with NIHSS (ρ = 0.354, p = 0.001) and mRS (ρ = 0.328, p = 0.010). ROC analysis identified cfDNA (>10,000 kilogenome equivalents/L) and hsCRP (>6 mg/L) as predictive thresholds for severe stroke and poor outcomes, with area under the curve (AUC) values of 0.79 and 0.71, respectively. Logistic regression indicated age > 60 years (OR 1.45, p = 0.041), cfDNA > 10,000 (OR 3.12, p = 0.027), hsCRP > 6 mg/L (OR 2.75, p = 0.039), and higher NIHSS (OR 1.23, p = 0.042) as significant predictors of poor neurological outcomes. These thresholds can guide early interventions, and the modest correlation coefficients reflect the multifactorial nature of stroke. This study uniquely proposes predictive thresholds for cfDNA and hsCRP in an Indian cohort, adding to the existing evidence on their clinical utility.

Conclusion

The study demonstrates that elevated cfDNA and hsCRP levels are significantly associated with stroke severity and poor outcomes in acute ischemic stroke patients. These biomarkers, alongside age and NIHSS score at admission, may serve as valuable tools in predicting prognosis and guiding early therapeutic interventions in stroke management. Future research should focus on evaluating the cost-effectiveness, feasibility, and integration of these biomarkers into routine clinical practice to optimize stroke care.

## Introduction

With an incidence rate of 172-198.8 per 100,000/year, stroke has become a major global health concern, accounting for over 10% of total deaths worldwide [1]. In India, the situation is particularly alarming, with a rising incidence driven by modern lifestyle changes, including an increased prevalence of hypertension, diabetes, sedentary behavior, unhealthy dietary habits, and an aging population [2]. Furthermore, limited public awareness and delayed medical attention exacerbate the burden, leading to significant morbidity and mortality. This underscores the urgent need to develop efficient and accessible diagnostic and prognostic tools to address this growing healthcare challenge.

The diagnosis of acute stroke, particularly acute ischemic stroke (AIS), which accounts for approximately 85% of all strokes, is primarily clinical, with neuroimaging modalities like computed tomography (CT) or magnetic resonance imaging (MRI) serving as crucial adjuncts [3,4]. Neuroimaging helps differentiate between ischemic and hemorrhagic strokes, assesses infarct size, and informs treatment decisions. However, in India, access to neuroimaging remains a significant barrier, especially in rural or resource-limited settings. Factors such as the high cost of imaging, limited availability of advanced portable neuroimaging technologies, and the shortage of trained radiologists pose substantial challenges [4,5]. Additionally, contraindications like renal dysfunction and allergies to contrast agents restrict neuroimaging use in certain patients [6]. These constraints emphasize the need for alternative diagnostic tools, such as blood biomarkers, that are cost-effective, easily deployable, and provide timely information on stroke severity and prognosis.

In this context, cell-free DNA (cfDNA) and high-sensitivity C-reactive protein (hsCRP) have emerged as promising biomarkers. cfDNA, released into the circulation during cell damage and apoptosis, correlates with infarct volume and stroke severity, highlighting its potential to reflect brain tissue injury [7,8]. For instance, prior studies have shown that cfDNA thresholds exceeding 10,000 kilogenome equivalents/L are linked to poor outcomes, underscoring its clinical relevance [8]. Meanwhile, hsCRP, a marker of systemic inflammation synthesized by the liver, has demonstrated strong associations with atherosclerosis, vascular events, and stroke outcomes [9]. Elevated hsCRP levels not only reflect inflammatory burden but also predict long-term functional outcomes in stroke patients, making it a reliable prognostic indicator [10].

cfDNA and hsCRP were prioritized over other potential biomarkers, such as S100B and interleukin-6, due to their demonstrated associations with infarct size, stroke outcomes, and the feasibility of their measurement in clinical practice [10]. Additionally, these markers align with the study's focus on acute ischemic stroke, a subset where inflammatory and cell-damage markers are particularly relevant. However, pre-existing conditions such as chronic inflammation or prior vascular events may influence biomarker levels, an aspect considered during analysis.

Given the potential utility of these markers, this study aimed to evaluate the significance of blood biomarkers such as cfDNA and hsCRP in assessing the severity and long-term prognosis of acute ischemic stroke. These findings will contribute to improving stroke management, especially in settings where neuroimaging is not readily accessible. Moreover, the findings could pave the way for cost-effectiveness studies and further research into integrating these biomarkers into routine clinical practice, particularly in scenarios where neuroimaging remains inaccessible or impractical.

## Materials and methods

This was a prospective clinical study conducted at a tertiary care teaching hospital, Pacific Medical University, catering to a mixed population from both urban and rural areas, which reflects a broad spectrum of patient characteristics seen in India. Fifty-four patients with acute ischemic stroke admitted to our institute hospital between April 2018 and April 2019 were included in the study. To ensure representativeness, patient demographics such as age, sex, and rural versus urban residence were documented.

Inclusion and exclusion criteria

The study included patients with the first episode of acute ischemic stroke, aged 18 to 90 years, with no history of incapacitating medical conditions. Stroke diagnosis was confirmed through brain imaging (CT or MRI) to establish ischemic pathology, ensuring accurate enrollment. Cases were classified based on etiopathogenesis, and only patients with confirmed ischemic stroke were included. Exclusion criteria were: trauma to the central nervous system, meningitis, encephalitis, systemic infections, hypertensive encephalopathy, tumors, migraine, post-cardiac arrest, drug overdose, organ failure, psychiatric syndromes, and shock. Patients presenting more than 12 hours after the onset of stroke symptoms were excluded. The 12-hour timeframe was chosen to minimize heterogeneity in outcomes caused by delayed presentation, as earlier interventions are more likely to impact biomarker levels and prognosis.

Sample size calculation

A detailed sample size calculation was performed using a significance level (p) of 0.05, and a power analysis revealed that a sample size of 54 would provide 80% power to detect clinically meaningful differences in biomarker levels. The calculation was based on an assumed effect size of 0.5, commonly used for moderate differences in clinical studies, and standard deviations derived from preliminary pilot data.

Laboratory procedure

In the hospital emergency room, a 5 mL venous blood sample was collected from each patient within six hours of hospital admission. This timeframe was chosen to standardize biomarker measurement relative to stroke onset, accounting for potential variability due to pre-hospital delays such as transport time and initial evaluation at referral centers. While six hours post-admission might not perfectly reflect symptom onset for all patients, it was considered a reasonable compromise to ensure consistency across the cohort while minimizing variability in cfDNA and hsCRP levels.

The blood samples were collected in ethylenediamine tetraacetic acid (EDTA) vials and centrifuged at 4°C for 20 minutes at 14,000 × g (relative centrifugal force) to separate plasma, ensuring plasma quality and cfDNA stability. To address deviations from these protocols (e.g., delays in centrifugation or fluctuations in temperature), a quality assurance protocol was followed. Deviated samples were flagged, and repeat sampling was performed if necessary. The detection limit for cfDNA quantification was 0.1 ng/mL, as validated through internal calibration with standards provided by the manufacturer.

cfDNA was extracted from a 1 mL plasma sample using the QIAamp circulating nucleic acid isolation protocol (QIAGEN N.V., Hilden, Germany). The cfDNA quantification was performed using real-time polymerase chain reaction (PCR) for the β-globin gene (QuantiNova; QIAGEN N.V.) on the ‘Rotor Gene Q’ PCR machine [11]. Primer sequences, quality control measures, and internal controls used during PCR are detailed in existing literature [12,13]. Replicates were performed for each measurement, and intra- and inter-assay variabilities were maintained below 5%, demonstrating measurement reliability. Inter-laboratory comparability for cfDNA quantification was ensured by cross-validating results against a reference laboratory during the pilot phase.

For hsCRP measurement, plasma samples were analyzed using the C-311 analyzer via the immunoturbidimetric method (F. Hoffmann-La Roche AG, Basel, Switzerland), with results expressed in mg/L. Quality control measures included duplicate assays and daily calibration of the analyzer using manufacturer-provided controls. Inter-laboratory standardization for hsCRP was achieved by adhering to international reference standards and comparing results with an external quality assurance program, ensuring comparability across settings.

Clinical assessment

Upon hospital admission, the severity of the stroke was assessed using the National Institutes of Health Stroke Scale (NIHSS). This scale measures neurologic deficits across eleven categories, and a score of ≤6 indicated less severe (mild to moderate) stroke, while a score of >15 represented severe stroke presentation. To track progression or improvement, NIHSS assessments were conducted at admission and again at discharge, allowing for a more comprehensive evaluation of clinical changes [14]. Three months post-stroke onset, neurological outcomes were assessed using the modified Rankin scale (mRS), a functional assessment tool for estimating neurologic deficits. Scores <3 were considered indicative of good neurologic outcomes, while scores ≥3 denoted poor recovery [15].

The clinical assessment and scoring were conducted by a clinician blinded to the laboratory test results, and the laboratory personnel processing the blood samples were also blinded to the clinical data to prevent potential bias. However, the use of NIHSS and mRS scales may have introduced potential inter-observer variability, particularly in subjective categories such as aphasia and functional disability. Efforts to mitigate this included ensuring assessments were performed by experienced clinicians trained in using these scales. Additionally, periodic cross-checks were conducted by a second blinded evaluator for a subset of patients to ensure consistency in scoring.

Challenges in maintaining blinding were minimal, but in instances where breaches occurred (e.g., inadvertent sharing of clinical data), the affected case was flagged, and subsequent evaluations were reviewed by an independent assessor. 

Statistical analysis

Data analysis was conducted using IBM SPSS Statistics for Windows, Version 25.0 (Released 2017; IBM Corp., Armonk, New York, United States). The normality of the data was assessed using the Shapiro-Wilk test. Non-parametric tests, such as the Mann-Whitney U test, were employed for comparisons of continuous variables (e.g., cf-DNA and hsCRP levels) when the data did not meet the assumption of normality, offering a robust alternative to parametric tests like the independent t-test. For normally distributed data, parametric tests were used as appropriate. Categorical data, including comorbidities and interventions, were analyzed using the Chi-square test.

Missing data were minimal and handled by exclusion from the specific analysis for the affected variable to avoid bias, as the missing cases were random. To control for the risk of type I errors due to multiple comparisons, a Bonferroni correction was applied where necessary, ensuring a more stringent significance threshold.

Spearman’s correlation coefficient (ρ) was used to evaluate the relationship between biomarker levels (cf-DNA and hsCRP) and stroke severity (NIHSS) as well as neurological outcomes (mRS at three months), with statistical significance set at a p-value < 0.05. Receiver operating characteristic (ROC) curves were generated to assess the prognostic value of cf-DNA and hsCRP for predicting poor neurological outcomes (mRS ≥ 3) at three months. The area under the curve (AUC) was reported with 95% confidence intervals (CI) to quantify the predictive accuracy.

Multiple logistic regression analysis was performed to identify independent predictors of poor neurological outcomes (mRS ≥ 3). Independent variables, including age, gender, cf-DNA levels, hsCRP levels, NIHSS score at admission, hypertension, diabetes, and smoking status, were selected based on prior evidence and clinical relevance. Multicollinearity diagnostics, such as variance inflation factor (VIF) calculations, were conducted to ensure no significant collinearity among predictors. Odds ratios (OR) with 95% CI were calculated, and statistical significance was defined as a p-value < 0.05.

Ethical consideration

The study was conducted following approval from the Ethics Committee of Pacific Medical University (approval number: PMU/PMCH/IEC/2018/32). Informed consent was obtained from each patient’s relative or attendant before inclusion, ensuring adherence to ethical standards for vulnerable populations. Patient autonomy was respected by clearly explaining the study's purpose, procedures, potential risks, and benefits to both patients (where possible) and their attendants. Relatives or attendants were encouraged to ask questions, and consent was obtained only after ensuring their understanding and voluntary agreement, thereby upholding ethical principles of autonomy and informed decision-making.

## Results

The median age of patients was 61 years, with a mean age of 61.6 ± 16.1 years. The study cohort comprised 68.5% male patients, with no significant difference in gender distribution between the mild-to-moderate stroke group (74.1% male) and the severe stroke group (77.3% male) (p = 0.795). The mean NIHSS score at admission was 12.9 ± 8.6, indicating a broad range of stroke severities, and the mean mRS score at three months was 2.56 ± 1.6, reflecting varied long-term outcomes. Diabetes was significantly more prevalent in the severe stroke group (27.3% vs. 12.5%, p = 0.036), and hypertension was also more common in the severe group (63.6% vs. 40.6%, p = 0.045), suggesting a potential pathophysiological link between these comorbidities and stroke severity. Although ischemic heart disease showed a numerical difference (18.2% in the severe group vs. 3.7% in the mild-to-moderate group), this difference was not statistically significant (p = 0.060). Regarding biomarkers, cf-DNA and hsCRP levels were significantly higher in the severe stroke group compared to the mild-to-moderate stroke group (mean cf-DNA 16,078.1 ± 8,483.6 vs. 7,432.5 ± 1,512.4 kilogenome equivalents/L, p < 0.0001; mean hsCRP 6.7 ± 4.3 vs. 2.5 ± 1.8 mg/L, p < 0.0001) (Table 1).

**Table 1:** Comparison of Baseline Characteristics of Study Population. Data given as frequency (percentage) except for Age, where it is given as Mean±SD

Variable	Overall (n = 54), n (%)	Mild to Moderate Stroke (n=32), n (%)	Severe Stroke (n=22), n (%)	p-Value
Age (years), mean±SD	61.6 ± 16.1	57.5 ± 15.7	65.8 ± 16.9	0.069
Gender				
Male	37 (68.5)	20 (74.1)	17 (77.3)	0.795
Female	17 (31.5)	7 (25.9)	5 (22.7)	
Comorbidities				
Diabetes mellitus	10 (18.5)	4 (12.5)	6 (27.3)	0.036
Hypertension	27 (50.0)	13 (40.6)	14 (63.6)	0.045
Ischemic heart disease	5 (9.2)	1 (3.7)	4 (18.2)	0.060
Hyperlipidemia	21 (38.9)	10 (37.1)	11 (50.0)	0.164
Smoking	12 (22.2)	5 (18.5)	7 (31.8)	0.159
Alcohol addiction	10 (18.5)	4 (12.5)	6 (27.3)	0.169

Patients who underwent thrombolysis had a mean cfDNA level of 8,976.7 ± 1,589.6 kilogenome eq/L and an hsCRP level of 3.8 ± 1.2 mg/L, while those who underwent thrombectomy exhibited lower mean cfDNA and hsCRP levels (7,591.8 ± 1,363.9 kilogenome eq/L and 4.1 ± 1.4 mg/L, respectively). In contrast, patients who did not receive any intervention showed significantly higher cfDNA (14,502.8 ± 2,629.4 kilogenome eq/L) and hsCRP (7.2 ± 2.1 mg/L) levels (p < 0.0001 for both). The higher biomarker levels in the no-intervention group may be associated with delayed presentation or contraindications to intervention, such as the presence of large infarcts or other clinical factors that prevent the use of thrombolysis or thrombectomy. Additionally, the differences between thrombolysis and thrombectomy could reflect variations in the mechanisms or timing of reperfusion. Thrombectomy may restore blood flow more rapidly and efficiently, potentially leading to lower biomarker levels, whereas thrombolysis, which works by dissolving clots, may involve a different mechanism that could result in a greater release of biomarkers from tissue injury. Moreover, patients with severe strokes (NIHSS > 15) had significantly higher mean cfDNA (16,078.1 ± 8,483.6 kilogenome eq/L) and hsCRP levels (6.7 ± 4.3 mg/L) compared to those with less severe strokes (NIHSS ≤ 6) (p < 0.0001), indicating that these biomarkers are associated with stroke severity. Similarly, patients with poor outcomes (mRS ≥ 3) had markedly elevated cfDNA (15,637.2 ± 8,990.6 kilogenome eq/L) and hsCRP (6.3 ± 4.1 mg/L) levels compared to those with good outcomes (mRS < 3) (p < 0.0001 and p = 0.001, respectively), further suggesting that these biomarkers are not only reflective of stroke severity but may also correlate with the extent of brain injury and long-term prognosis. Therefore, cfDNA and hsCRP levels could potentially serve as biomarkers for prognostication in acute ischemic stroke (Table 2).

**Table 2:** Comparison of Biomarkers (cfDNA and hsCRP) Between Intervention/Outcome. NIHSS: National Institutes of Health Stroke Scale; cfDNA: cell-free DNA; hsCRP: high-sensitivity C-reactive protein; mRS: modified Rankin Scale.

Variables	cfDNA (kilogenome eq/L), mean ± SD	hsCRP (mg/L), mean ± SD
Intervention		
Thrombolysis (n=15)	8976.7 ± 1589.6	3.8 ± 1.2
Thrombectomy (n=11)	7591.8 ± 1363.9	4.1 ± 1.4
No Intervention (n=28)	14502.8 ± 2629.4	7.2 ± 2.1
p-value	<0.0001	<0.0001
NIHSS at admission		
Mild to Moderate Stroke (n=32)	7432.5 ± 1512.4	2.5 ± 1.8
Severe Stroke (n=22)	16078.1 ± 8483.6	6.7 ± 4.3
p-value	<0.0001	<0.0001
mRS at 3 months		
Good Outcome (n=26)	6877.8 ± 1352.8	3.2 ± 1.5
Poor Outcome (n=28)	15637.2 ± 8990.6	6.3 ± 4.1
p-value	<0.0001	0.001

The Spearman correlation analysis revealed significant positive correlations between both cf-DNA and hsCRP levels with stroke severity and functional outcomes. cf-DNA levels were moderately associated with stroke severity, as reflected by a correlation coefficient of ρ = 0.222 (p = 0.040) for NIHSS scores, and showed a stronger positive correlation with 3-month functional outcomes, with ρ = 0.396 (p = 0.002) for mRS scores. This suggests that higher cf-DNA concentrations are linked to increased stroke severity and poorer long-term outcomes. Similarly, hsCRP levels demonstrated a significant positive correlation with stroke severity (ρ = 0.354, p = 0.001) and with 3-month mRS scores (ρ = 0.328, p = 0.010), indicating that elevated hsCRP levels are associated with both more severe strokes and worse functional outcomes (Table 3).

**Table 3:** Correlation Between Biomarkers (cfDNA and hsCRP) and Stroke Severity and Outcomes. NIHSS: National Institutes of Health Stroke Scale; cfDNA: cell-free DNA; hsCRP: high-sensitivity C-reactive protein; mRS: modified Rankin Scale.

Biomarker	Spearman Correlation Coefficient (ρ), p-value	
	NIHSS	mRS
cfDNA	0.222, 0.040	0.396, 0.002
hsCRP	0.354, 0.001	0.328, 0.010

**Figure 1:**
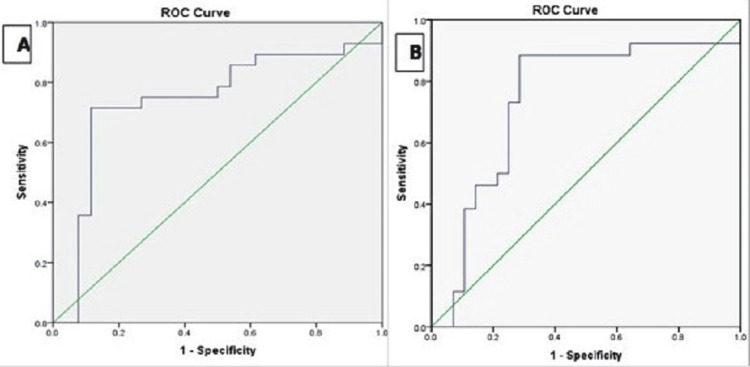
(A) ROC curve for cf-DNA level and mRS three-month score; (B) ROC curve for hsCRP level and mRS three-month score. ROC: receiver operating characteristic; cfDNA: cell-free DNA; hsCRP: high-sensitivity C-reactive protein; mRS: modified Rankin Scale

The analysis of biomarker thresholds revealed distinct patterns in sensitivity and specificity for predicting poor neurological outcomes. For cf-DNA, a threshold of >10,000 kilogenome equivalents/L demonstrated high sensitivity (82%) and specificity (78%), with an AUC of 0.79, indicating its strong predictive accuracy. This suggests that cf-DNA is closely associated with tissue injury in acute ischemic stroke, enabling it to effectively identify patients at risk of poor outcomes. In contrast, the hsCRP threshold of >6 mg/L prioritized specificity (75%) over sensitivity (71%), yielding an AUC of 0.71. While hsCRP was less predictive overall compared to cf-DNA, it provided valuable insights into the inflammatory response associated with stroke severity. Notably, some overlap in hsCRP thresholds was observed between fair and poor outcomes, reflecting the influence of chronic inflammation and comorbid conditions (Figure 1). 

In our study, both cf-DNA and hsCRP levels were significantly correlated with stroke severity, as measured by the NIHSS. Elevated cf-DNA levels (ρ = 0.222, p = 0.040) and hsCRP levels (ρ = 0.354, p = 0.001) demonstrated a strong association with increased stroke severity and poorer functional outcomes. This finding aligns with previous studies showing that these biomarkers can reflect ischemic injury and inflammation, which are critical in determining stroke severity. However, the correlation coefficients were modest (ρ between 0.222 and 0.396), indicating that while these biomarkers are valuable in assessing stroke severity, they should be used in conjunction with other clinical factors for comprehensive risk assessment. Traditional risk factors such as hypertension, diabetes, and smoking did not show statistically significant associations with poor outcomes in our cohort, although the p-values for hypertension (p = 0.067) and diabetes (p = 0.088) were close to 0.05, suggesting a potential relationship that could be observed in a larger cohort. The logistic regression analysis revealed that elevated cf-DNA levels (>10,000 kilogenome eq/L), age over 60 years, and elevated hsCRP levels (>6 mg/L) were significantly associated with poor neurological outcomes (mRS ≥ 3), with ORs of 3.12, 1.45, and 2.75, respectively. The narrow confidence intervals for these significant variables (cf-DNA: 95% CI 1.75-5.56; hsCRP: 95% CI 1.42-4.58) suggest that these associations are robust. The cf-DNA threshold of >10,000 kilogenome eq/L emerged as a strong predictor of poor outcomes, but its clinical applicability in different populations needs further validation. Although hsCRP showed a lower OR (2.75), it may serve as an adjunct to cf-DNA rather than a standalone marker in predicting stroke outcomes (Table 4).

**Table 4:** Logistic Regression Analysis Predicting Poor Neurological Outcome (mRS ≥ 3). NIHSS: National Institutes of Health Stroke Scale; cfDNA: cell-free DNA; hsCRP: high-sensitivity C-reactive protein; mRS: modified Rankin Scale.

Variable	Odds Ratio (OR)	95% Confidence Interval (CI)	p-value
Age > 60 years	1.45	1.10 - 2.10	0.041
Gender (Male)	1.15	0.50 – 2.62	0.727
cf-DNA > 10,000	3.12	1.75 - 5.56	0.027
hsCRP > 6 mg/L	2.75	1.42 - 4.58	0.039
NIHSS score at admission	1.23	1.10 – 1.30	0.042
Hypertension	1.26	0.85 - 1.70	0.067
Diabetes	1.18	0.90 - 1.65	0.088
Smoking	1.16	0.75 - 1.48	0.155

## Discussion

The findings of our study highlight the critical role of blood biomarkers, specifically cfDNA and hsCRP, in assessing stroke severity and predicting outcomes in acute ischemic stroke patients. Elevated cfDNA levels significantly correlated with both NIHSS scores (ρ = 0.222, p = 0.040) and mRS scores (ρ = 0.396, p = 0.002), suggesting that higher cfDNA concentrations are indicative of increased stroke severity and poorer functional outcomes. This aligns with previous research by Tiwari et al., which demonstrated that plasma DNA concentration correlates with stroke severity and can be used as a prognostic marker in emergency settings [16]. The release of cfDNA into the bloodstream is thought to occur due to cellular and nuclear damage following ischemic injury, supporting the notion that cfDNA may serve as a marker of tissue damage in acute ischemic stroke [17,18].

Our analysis revealed that patients with severe strokes (NIHSS > 15) exhibited significantly higher cfDNA levels (16,078.1 ± 8,483.6 kilogenome eq/L) compared to those with less severe strokes (NIHSS ≤ 6) (7432.5 ± 1512.4 kilogenome eq/L, p < 0.0001). This finding corroborates earlier studies by O'Connell et al. [19] and Roth et al. [20], indicating that cf-DNA levels can reflect infarct volume and the immune response to cerebral ischemia, further establishing its potential as a biomarker for predicting stroke outcomes.

Similarly, hsCRP levels demonstrated a significant correlation with both stroke severity and functional outcomes, with correlation coefficients of ρ = 0.354 (p = 0.001) for NIHSS and ρ = 0.328 (p = 0.010) for mRS. Our results support findings by Chaudhuri et al. [21], Wang et al. [22], and Glickman et al. [23], who reported elevated hsCRP levels in ischemic stroke patients, correlating with the risk of poor outcomes and mortality. hsCRP, an acute-phase reactant synthesized by the liver in response to inflammation, plays a pivotal role in the pathogenesis of atherosclerosis by promoting thrombotic events and inflammatory responses [24,25]. The presence of chronic inflammation associated with traditional risk factors such as hypertension and diabetes may exacerbate neuronal injury in ischemic stroke, underscoring the importance of inflammatory markers like hsCRP in understanding stroke pathophysiology [25].

The ROC curve analysis further reinforced the prognostic value of cfDNA and hsCRP, with AUC values of 0.79 for cf-DNA and 0.71 for hsCRP, indicating their utility in predicting poor outcomes, with optimal thresholds established at >10,000 kilogenome equivalents/L for cf-DNA and >6 mg/L for hsCRP. These thresholds align with prior studies by Singleton et al. [26] and Dias et al. [27], that have emphasized the relevance of these biomarkers in stratifying stroke risk and predicting outcomes.

Furthermore, our logistic regression analysis identified age, cfDNA, hsCRP, and NIHSS scores at admission as significant predictors of poor neurological outcomes, with ORs of 1.45, 3.12, 2.75, and 1.23, respectively. This underscores the multifactorial nature of stroke pathology and highlights the necessity for individualized risk assessments in stroke management. However, it is important to note that while these biomarkers are statistically significant, their modest correlation coefficients (ρ = 0.222-0.396) suggest that they may not be strong standalone predictors. The predictive value of cfDNA and hsCRP could be enhanced by incorporating them into a more comprehensive risk stratification model that includes other clinical parameters, such as neuroimaging findings (e.g., infarct volume), genetic markers, or established stroke severity scores like the NIHSS. Combining biomarkers with imaging data could provide a more accurate prediction of outcomes, helping clinicians tailor therapeutic approaches for individual patients [28].

Although the thresholds for cfDNA and hsCRP identified in the current study appear promising, further validation in larger, multicenter cohorts is necessary to determine their consistency across diverse populations. Additionally, external validation of these thresholds, particularly in non-acute settings or across different ethnic groups, is crucial for their broader application in clinical practice. The use of cfDNA and hsCRP could also be explored beyond acute ischemic stroke, potentially extending to hemorrhagic stroke or other neurological conditions, where these biomarkers may offer insights into disease pathophysiology and prognosis.

The AUC values for both cfDNA and hsCRP suggest good to fair predictive capacity. However, there may be an overlap in their predictive power with other established biomarkers or clinical scores, which could limit their clinical utility as standalone tools. For example, clinical factors such as age, stroke subtype, and comorbid conditions could also influence stroke severity and outcomes, and must be carefully considered when applying these biomarkers in clinical settings. Further research is needed to determine how best to integrate cfDNA and hsCRP with other clinical, imaging, and genetic markers to improve stroke risk stratification.

In addition to these considerations, the methodological challenges in estimating cfDNA levels, including variability in sample handling, timing of measurements, and assay standardization, should not be overlooked. Standardizing these variables will be crucial for ensuring the reproducibility and reliability of cfDNA as a clinical biomarker.

Future research could explore the combined utility of cfDNA and hsCRP with advanced imaging biomarkers such as diffusion-weighted imaging or MRI biomarkers to enhance early stroke detection and prognosis. Furthermore, the role of cfDNA and hsCRP in post-stroke recovery could be investigated, as these biomarkers may help track the resolution of inflammation and tissue repair.

Limitations

This study has several limitations that should be considered when interpreting the findings. First, the small sample size of 54 patients likely reduces the statistical power, particularly for subgroup analyses, such as comparisons between stroke severity groups or ROC threshold determination, potentially leading to unreliable or less precise results. Second, cultural, genetic, and environmental factors specific to the Indian study population may influence biomarker levels, further limiting the generalizability of the findings to other populations. For example, dietary habits, prevalence of comorbidities like diabetes or hypertension, and genetic predispositions could contribute to variations in cfDNA and hsCRP levels.

The timing of blood collection within six hours post-admission, though standardized, requires further contextualization, as evidence suggests that cfDNA and hsCRP levels may fluctuate significantly in the acute phase of stroke. A more nuanced understanding of how these biomarkers vary with the progression of ischemic injury could provide deeper insights. Additionally, the cross-sectional design precludes the ability to capture longitudinal changes in biomarker levels over time and their dynamic relationships with stroke recovery, which longitudinal studies would address more effectively.

Moreover, focusing solely on cfDNA and hsCRP may overlook the contributions of other clinically relevant markers such as D-dimer or fibrinogen, which could complement these biomarkers in refining stroke severity assessment and risk stratification. Expanding future research to include these additional variables could enhance the predictive utility of biomarker panels. Finally, conducting the study in a single tertiary care center limits its applicability to broader and more diverse settings, highlighting the need for multicenter studies to validate these findings.

## Conclusions

Our findings contribute to the growing body of literature supporting the use of cfDNA and hsCRP as biomarkers for assessing stroke severity and predicting outcomes. Integrating these biomarkers into clinical practice could enhance prognostic accuracy and lead to more tailored therapeutic strategies, such as targeted anti-inflammatory therapies or risk stratification models. For instance, identifying patients with elevated cfDNA or hsCRP levels could prioritize them for intensive monitoring or early intervention to prevent secondary complications. Future research should delve into specific areas such as the role of cfDNA in apoptosis or its association with oxidative stress pathways, which could further elucidate the biological mechanisms underpinning stroke pathology. Additionally, studies should explore the utility of these biomarkers across different stroke subtypes, including hemorrhagic or cryptogenic strokes, to assess their broader applicability. Validating these findings in larger, multicentric cohorts will be essential to ensure generalizability and establish robust clinical guidelines for integrating these biomarkers into routine stroke care.
